# Oral Microcapsule Chromocolonoscopy With Patent Blue V Improves Adenoma Detection Safely and Effectively

**DOI:** 10.1002/ueg2.70067

**Published:** 2025-07-05

**Authors:** Berenice Schulte, Georg H. Waetzig, Johannes Bethge, Konrad Aden, Claudio C. Conrad, Eva‐Maria Theismann, Julia K. Keppler, Therese Ruhmlieb, Karin Schwarz, Stefan Schreiber, Mark Ellrichmann

**Affiliations:** ^1^ Interdisciplinary Endoscopy Department of Internal Medicine I University Hospital Schleswig‐Holstein Campus Kiel Kiel Germany; ^2^ Institute of Clinical Molecular Biology Christian‐Albrechts‐University Kiel and University Hospital Schleswig‐Holstein, Campus Kiel Kiel Germany; ^3^ CONARIS Research Institute AG Kiel Germany; ^4^ Division of Food Technology Christian‐Albrechts‐University Kiel Kiel Germany; ^5^ Department of Agrotechnology and Food Sciences Laboratory of Food Process Engineering Wageningen University & Research Wageningen the Netherlands; ^6^ Department of Internal Medicine University Hospital Oldenburg Oldenburg Germany

**Keywords:** adenoma detection rate, chromocolonoscopy, colorectal carcinoma, inflammatory bowel diseases, microcapsules, patent blue V, pH‐dependent release, polyp detection, shellac coating, ulcerative colitis

## Abstract

**Background and Objective:**

Chromocolonoscopy significantly improves polyp/adenoma detection rates (PDR/ADR). However, its integration into routine clinical practice is hindered by its cumbersome mode of application. The objective was to develop an oral nutritional grade delivery system and to assess its efficacy for colonic release, mucosal staining and PDR/ADR as a proof of concept.

**Methods:**

Food‐grade shellac microcapsules releasing 87.5 mg of patent blue V (PBM) pH‐ and time‐dependently were used in 35 volunteers receiving diagnostic colonoscopy either due to a positive fecal occult blood test or surveillance in inflammatory bowel disease. Six capsules were administered p.o. during the bowel preparation (Klean‐Prep). Mucosal staining was assessed in total and per segment using a five‐point grading scale. PDR and ADR were evaluated and compared to a propensity score‐matched comparison cohort in a 1:3 ratio.

**Results:**

In the PBM cohort, 97.1% (34/35) achieved an optimal to acceptable staining quality (SQ) score of ≥ 8, with a mean total score of 13.4 ± 2.9. PDR was significantly higher in the PBM group at 62.8% compared to 42.9% in the comparison group (CG; *p* = 0.04). ADR showed no significant differences (*p* = 0.06). The use of PBM resulted in a significantly increased number of detected polyps and adenomas per colonoscopy compared with CG (polyps: PMB = 1.1 ± 1.1 vs. CG = 0.6 ± 0.8, *p* = 0.02; adenomas: PBM = 0.8 ± 0.9 vs. CG = 0.3 ± 0.5; *p* = 0.02).

**Conclusion:**

The novel PBM demonstrated uniform mucosal staining when utilized in chromocolonoscopy. Delayed‐release patent blue V appears to be a safe and effective alternative to dye‐spray techniques and existing oral chromoendoscopy modalities.

## Summary

- Established knowledge on this subject
  ◦ Chromocolonoscopy improves the visualization and differentiation of mucosal structures, hence increases the polyp and adenoma detection rates, and is especially recommended for high‐risk individuals with colorectal carcinoma or patients with UC undergoing surveillance colonoscopy.
  ◦ Dye‐based chromocolonoscopy employing spraying catheters is inconvenient to use in the daily routine; the recently approved oral formulation of methylene blue MMX capsules contains the controversial absorptive dye methylene blue.
  ◦ Patent blue V is a well‐established and safe food‐dye (E131) endorsed by the European Food Safety Authority.
  ◦ Food‐grade shellac can be used for encapsulation.
- The significant and new findings of this case series
  ◦ Development, stability testing and production of microcapsules formulated by spray‐coating cellulose matrix cores containing patent blue V with two layers of food grade shellac and an interlayer of citric acid for pH buffering.
  ◦ Proof of concept of the pH‐dependent burst release.
  ◦ Effective staining of the colonic mucosa with patent blue V microcapsules.
  ◦ Elevated polyp detection rates by chromocolonoscopy with patent blue V microcapsules versus propensity score matching comparison population.
  ◦ No significant rise of adverse events according to the use of patent blue V microcapsules.
  ◦ No prolongation of the examination times during chromocolonoscopy after self‐administered staining with patent blue V microcapsules.

## Graphical abstract

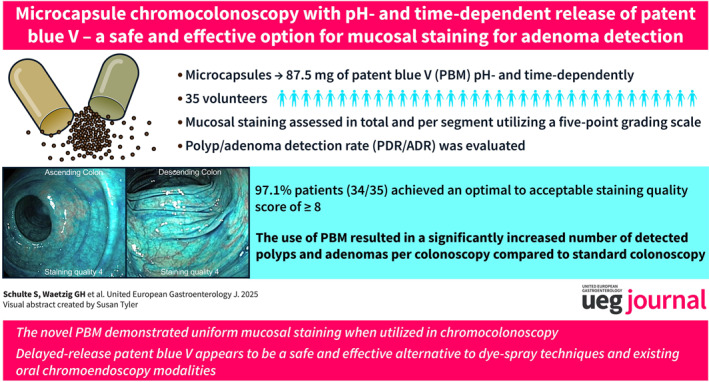

**Abbreviations**
AC: ascending colon
ADI: acceptable daily intake
ADR: adenoma detection rate
AE: adverse event(s)
AI: artificial intelligence
AMR: adenoma miss rate
ASA: American Society of Anesthesiologists
BBPS: Boston Bowel Preparation Scale
CADe: computer assisted detection
CADx: computer assisted differentiation
CRC: colorectal cancer
DC: descending colon
DGVS: Deutsche Gesellschaft für Verdauungs‐und Stoffwechselerkrankungen
EFSA: European Food Safety Authority
ESGE: European Society of Gastrointestinal Endoscopy
HDWL(E): High‐definition white light (endoscopy)
IBD: inflammatory bowel diseases
MB: methylene blue
MB‐MMX: methylene blue MMX tablets
PB: patent blue V
PBM: patent blue V microcapsules
PDR: polyp detection rate
PSM: propensity score matching
SD: standard deviation
SMDs: standardized mean differences
SQ: staining quality
SR: sigmoid/rectum
TC: transverse colon
UC: ulcerative colitis
WLI: white light imaging

## 1 Introduction

The European Society of Gastrointestinal Endoscopy (ESGE) has established quality criteria for screening and diagnostic colonoscopy, serving as a benchmark for endoscopic practice and ensuring optimal patient outcomes. The polyp (PDR) and adenoma detection rates (ADR) have been defined as a key criteria for ultimately reducing colorectal cancer (CRC) through early detection and prevention [1]. Despite the procedure's high diagnostic accuracy, a recent meta‐analysis evaluating 43 randomized controlled trials with more than 15,000 tandem colonoscopies exhibited an adenoma miss rate (AMR) of 26%. The respective risk of an interval colorectal cancer was estimated between 1.8% and 4.0% [2].

Therefore, enhancing ADR stands as a crucial imperative in CRC prevention with various technical modalities [3]. Chromoendoscopy improves the visualization and differentiation of mucosal structures by applying either digital contrast and color enhancements (dye‐less, e.g., narrow band imaging (NBI)) or direct mucosal staining with different dyes (dye‐based) [4], which is still considered as the gold standard. Beyond significantly increasing ADR in screening colonoscopy [3, 5], chromocolonoscopy is particularly advocated in the surveillance of CRC in inflammatory bowel diseases (IBD), especially ulcerative colitis (UC) [4]. Direct application of dyes enhances visualization of subtle architectural changes of the mucosa, thereby improving the detection rate of dysplasia in UC. Both clinical trials and real‐world studies show that chromoendoscopy outperforms white‐light endoscopy in the detection of dysplasia, which is consistent regardless of the endoscopist's level of experience or the availability of high‐resolution endoscopic equipment [6, 7].

Although dye‐based chromoendoscopy has demonstrated superior efficacy in dysplasia and cancer detection, its widespread adoption remains limited. This is primarily attributed to the heightened procedural demands in terms of time, sedation, and cleaning, coupled with elevated costs and advanced skills to achieve uniform mucosal staining. To overcome these problems, an oral administration of methylene blue tablets has recently been approved (Methylene Blue MMX; MB‐MMX, Cosmo Pharmaceuticals, Dublin, Ireland) and showed an increase in ADR of approximately 9% compared to placebo in a phase III trial [8]. Former studies provide an indication that the incorporation of MB into epithelial and other cells might have a risk of causing DNA damage on a micro‐level [9, 10]. Nevertheless, after a single use in routine clinical practice, there was no evidence of a short‐term clinical adverse effect from this DNA damage [11].

Considering these potential adverse effects, staining with a food‐grade dye appears to offer a safer alternative. Patent blue V (PB), a well‐established food‐dye (E131), exhibits minimal absorption, lacks metabolic conversion and has been endorsed by the European Food Safety Authority (EFSA) with an acceptable daily intake (ADI) of 5 mg/kg/day [12]. In our previous research, our group developed shellac‐based microcapsules designed for prolonged colonic drug release [13, 14]. A double layer of shellac, which was pH‐buffered by a citric acid intermediate layer, provides a highly reliable mechanism for pH‐dependent, prolonged release of PB into the ileum and colon [13, 14, 15].

Building upon this unique release principle, the aim of this investigation was to assess shellac food‐grade microcapsules suitable for oral administration, incorporating PB as a proof of principle for their performance in ileo‐colonic release, mucosal staining, and their effectiveness in PDR and ADR.

## 2 Material and Methods

### 2.1 Study Overview

Microcapsules with a diameter of 0.35 mm were formulated by spray‐coating cellulose matrix cores containing PB with two layers of food grade shellac and an interlayer of citric acid for pH‐buffering (PBM, PB microcapsules, manufactured by the Department of Food Technology, Christian‐Albrechts‐University Kiel, Kiel, Germany). The architecture of three layers provides a sharp pH‐dependent release profile. Between December 2018 and March 2022, 35 patients scheduled for either diagnostic colonoscopy or a UC surveillance colonoscopy volunteered to receive a chromoendoscopy using six PBM alongside their standard bowel preparation. This was given under compassionate use criteria and was classified as a nutritional intervention.

The evaluation of this case series received approval from the Ethics Committee of the Medical Faculty at Christian‐Albrechts‐University, Kiel, Germany (Reference Numbers: D439/15 and D579/23). Written informed consent was obtained from all participants. All procedures were conducted at the Interdisciplinary Endoscopy Unit of the University Hospital Schleswig‐Holstein, Campus Kiel, Germany.

The primary endpoint of the evaluation was defined as the staining score of the respective bowel segments, serving as proof‐of‐concept testing for the new formulation. Secondary endpoints included PDR, ADR, number and size of polyps, adverse events, and examination times. Patients were matched in a 1:3 ratio with individuals undergoing either diagnostic or surveillance colonoscopy using propensity score matching.

### 2.2 In‐ and Exclusion Criteria

Two patient cohorts were selected: (i) individuals referred for diagnostic colonoscopy following a positive fecal occult blood test (FOBT) [16] and (ii) patients with UC in clinical remission who were scheduled for surveillance colonoscopy [17]. Exclusion criteria included pregnancy, American Society of Anesthesiologists (ASA) risk classes III‐V, inadequate bowel preparation (Boston Bowel Preparation Score [BBPS] < 2 per segment), incomplete colonoscopy (cecum not reached) and inability to provide informed consent.

### 2.3 Shellac‐Coated Patent Blue V Microcapsules and Application

**FIGURE 1:**
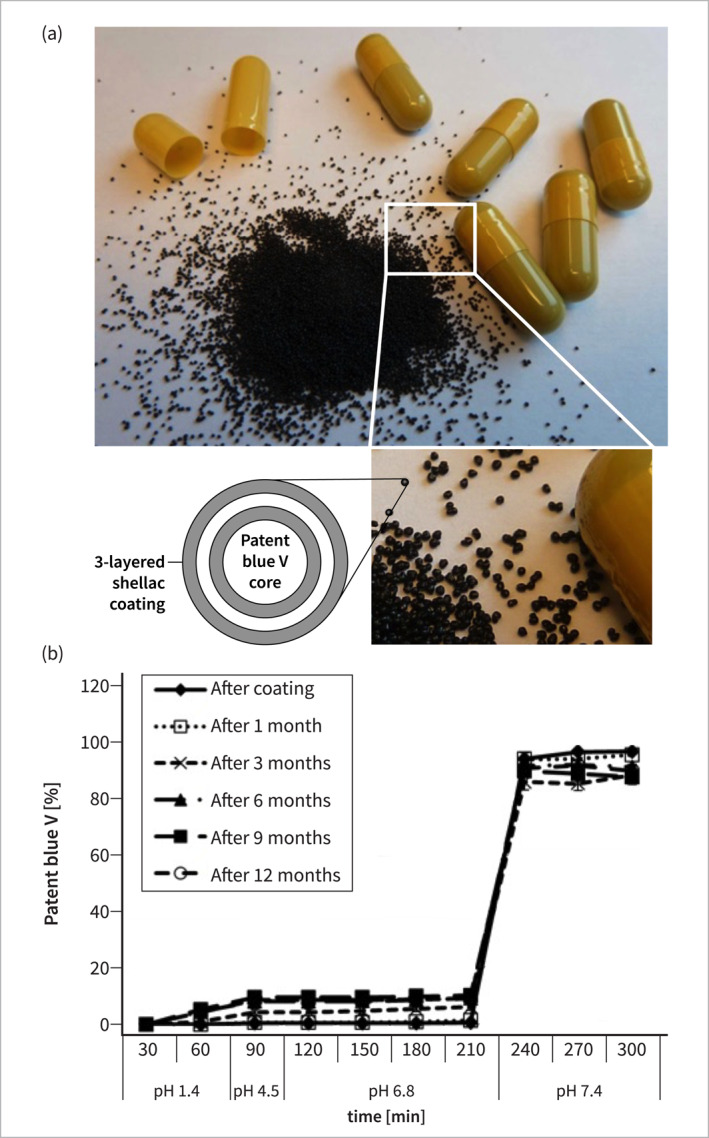
(a) Composition of patent blue V microcapsules (PBM) with 3‐layered coating, two layers of shellac pH‐buffered by a citric acid intermediate; (b) pH‐dependent release profile of PBM and stability assessment immediately after coating and after 1, 3, 6, 9 and 12 months of storage.

To deliver PB to the colonic mucosa, we employed microcapsules with a triple coating system and two layers of food‐grade shellac enclosing citric acid to ensure pH‐buffering and subsequent pH‐dependent release within the terminal ileum [14] (Figure 1). Gelatin capsules (size 0) filled with 87.5 mg PBM were used for self‐administration. Dissolution of the PBM and stability under long‐term storage conditions at room temperature were tested as described [14] (Figure 1).

Patients in the PBM group underwent bowel preparation using a split‐dose regimen in adherence to the manufacturer's patient information. On the day before colonoscopy, patients consumed 3 L of Macrogol 3350 (Klean‐Prep, Norgine GmbH, Wettenberg, Germany), accompanied by the ingestion of 2 PBM gelatin capsules each after the second and third liter. One liter followed by 2 more capsules was administered the next morning. The interval between the ingestion of the final capsules and the colonoscopy was approximately 4 h (Table S1). The timing and sequence of PBM capsule administration were designed to optimize bowel cleansing and mucosal staining, ensuring effective colonic delivery. After 2 L of macrogol for initial cleansing, PBM capsules were given stepwise during ongoing preparation to achieve gradual uniform dye release. A four‐hour interval between final capsule ingestion and colonoscopy allowed sufficient small bowel transit and colonic release.

Control patients underwent identical preparation without PBM. Klean‐Prep was selected for use with the chromocapsule as the only available isoosmotic solution, preserving the osmotic balance to maintain capsule integrity and prevent premature release, ensuring targeted delivery and diagnostic accuracy [18, 19].

#### 2.3.1 Colonoscopy

High‐definition white light colonoscopies (HDWLE) were performed under conscious sedation using Propofol (Propofol‐Lipuro 10 mg/mL, Braun Melsungen AG, Melsungen, Germany) upon patients’ request, with continuous monitoring throughout the procedure in accordance with current guidelines [20]. All procedures were conducted using Olympus equipment, including the EVIS X1 processor and EXERA III with CF‐190 colonoscopes (Olympus, Tokyo, Japan). All colonoscopies comprised in this study were performed by two highly experienced endoscopists (ME and JB), each with experiences exceeding 3000 diagnostic/surveillance colonoscopies. HDWL mode was used exclusively during withdrawal from the cecum in all procedures. NBI was applied solely for the characterization of polyps following detection under HDWL. Quality criteria for colonoscopy [1] were recorded.

### 2.4 Staining

The PBM staining quality (SQ) of the colon was evaluated by scoring each specified region of interest: the ascending colon (AC), transverse colon (TC), descending colon (DC), and sigmoid/rectum (SR). The total staining score was determined by summing the scores of all four segments. The staining scores were defined as: 0, no staining; 1, traces of staining (poor traces in colonic mucosa); 2, detectable staining (at least 25% of colonic mucosa stained); 3, acceptable staining (at least 50% of colonic mucosa stained); 4, good staining (at least 75% of colonic mucosa stained); 5, overstained (relevant patches of colonic mucosa overstained and not assessable after suction of colored fluid) [21]. The SQ for each patient was defined as a total score of 8 or higher with no signs of overstaining (score 5). The recorded images of the endoscopies were reviewed by an independent, experienced endoscopist (CCC, experience > 3000 diagnostic/surveillance colonoscopies) blinded to any additional information of the respective patient to verify concordance with the investigator's interpretation regarding the quality of PBM staining in each bowel segment. Additionally, the reviewer assessed whether the areas where polyps were identified were stained or unstained.

### 2.5 Statistics

A propensity score matching (PSM) analysis was performed to compare patients with positive fecal occult blood test undergoing diagnostic colonoscopy and patients with UC undergoing surveillance colonoscopy, with or without the use of PBM. PSM was conducted separately for the diagnostic and surveillance groups. Patients who underwent colonoscopy with PBM were matched in a 1:3 ratio to those without PBM. The propensity scores were calculated using a logistic regression model based on the following matching criteria: age (continuous variable), sex (categorical: male or female), indication for colonoscopy (categorical: diagnostic/surveillance colonoscopy), and quality of bowel preparation (continuous variable). Nearest‐neighbor matching without replacement was applied, and a caliper width was used to reduce mismatches and optimize balance between the groups. After matching, the balance of baseline characteristics was evaluated using standardized mean differences (SMD) with a threshold of < 0.1 indicating good balance. The final matched cohort demonstrated comparable baseline characteristics between the groups. A 1:3 propensity score was used to optimize statistical power, enhance precision, make full use of available data, and maintain appropriate covariate balance. From a clinical perspective, the 1:3 ratio also reflects real‐world practice, in which chromoendoscopy is performed less frequently due to its greater time and resource demands.

Descriptive statistics are presented as mean ± standard deviation (SD) unless otherwise stated. Comparative analyses were performed using the Mann‐Whitney test for nonparametric comparisons or a contingency table with calculation of Fisher's exact test, as appropriate. A *p* value of < 0.05 was considered statistically significant. All statistical analyses were performed using GraphPad Prism Version 10 (GraphPad Software, La Jolla, CA, USA).

## 3 Results

### 3.1 Quality Control of Patent Blue V Microcapsules

In vitro experiments demonstrated a consistent stability of PBM across designated pH levels of 1.4, 4.5, and 6.8. At pH 7.4, a characteristic burst release pattern of PB was observed that was sustained throughout 12 months at ambient temperature, affirming the formulation's enduring stability (Figure 1).

### 3.2 Patient Characteristics

**FIGURE 2:**
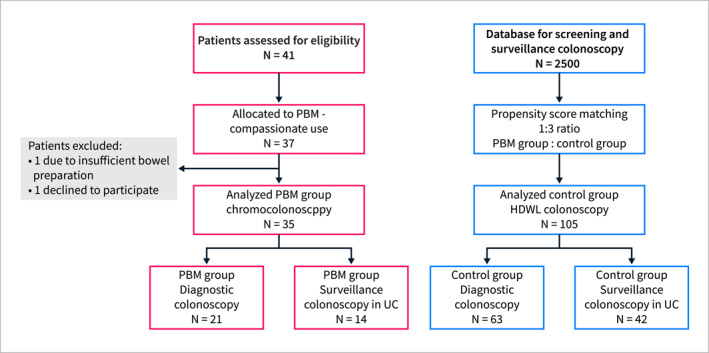
Flow chart of the study. This flow chart shows the composition of the two analyzed groups. In both arms, NBI was used when required. Both groups received the same bowel preparation and were examined by the same group of endoscopists.

41 patients were assessed for eligibility. 37 patients volunteered to participate in the case series under “compassionate use criteria”. Two patients were excluded from further analysis: one due to inadequate preparation for colonoscopy (BBPS = 2), another one withdrew the consent to participate. Ultimately, the PBM group consisted of 35 patients (21 diagnostic colonoscopies; 14 surveillance colonoscopies in UC), who were matched in a 1:3 ratio with 105 control group patients (63 diagnostic and 42 surveillance colonoscopies) based on their respective propensity scores (Figure 2). The key quality criteria showed no significant disparities between the PBM and control groups, as summarized in Table 1.

**TABLE 1:** Patient characteristics and quality criteria of colonoscopy.

	Total			Diagnostic colonoscopy 1:3 ratio propensity score matching			Surveillance in ulcerative colitis 1:3 ratio propensity score matching		
	Patent blue V microcapsules (PBM)	Control	*p* value	Patent blue V microcapsules (PBM)	Control	*p* value	Patent blue V microcapsules (PBM)	Control	*p* value
Patient characteristics									
Number of patients [*N*]	35	105		21	63		14	42	
Female [*N*] (%)	13 (37.1)	39 (37.1)	> 0.9	10 (47.6)	34 (53.9)	0.6	7 (50)	22 (52.4)	0.9
Age [years]	61.2 ± 7.8	60.4 ± 6.4	0.2	63 ± 8.1	61.9 ± 5.7	0.9	58.6 ± 8.1	58.1 ± 6.7	0.1
Indication of colonoscopy [*N*] (%)									
Diagnostic colonoscopy (positive FOBT)	21 (60)	63 (60)	> 0.9						
Surveillance colonoscopy in ulcerative colitis	14 (40)	42 (40)	> 0.9						
Quality criteria of colonoscopy									
Boston Bowel Preparation Score [median] (25%/75%‐percentile)	9 (8/9)	8 (8/9)	> 0.9	9 (8/9)	8 (8/9)	0.7	8 (9/9)	8 (8/9)	0.9
Net withdrawal time [min] (mean ± SD)	8.8 ± 1.8	8.3 ± 1.9	0.1	8.9 ± 2.3	8.5 ± 1.9	0.1	8.7 ± 1.3	8.1 ± 1.9	0.1
Total procedure time [min] (mean ± SD)	17.4 ± 5.4	18.2 ± 5.6	0.3	16.4 ± 6.0	17.7 ± 5.2	0.3	18.4 ± 4.8	18.7 ± 6	0.3

Abbreviation: FOBT, fecal occult blood test.

### 3.3 Staining Quality

**FIGURE 3:**
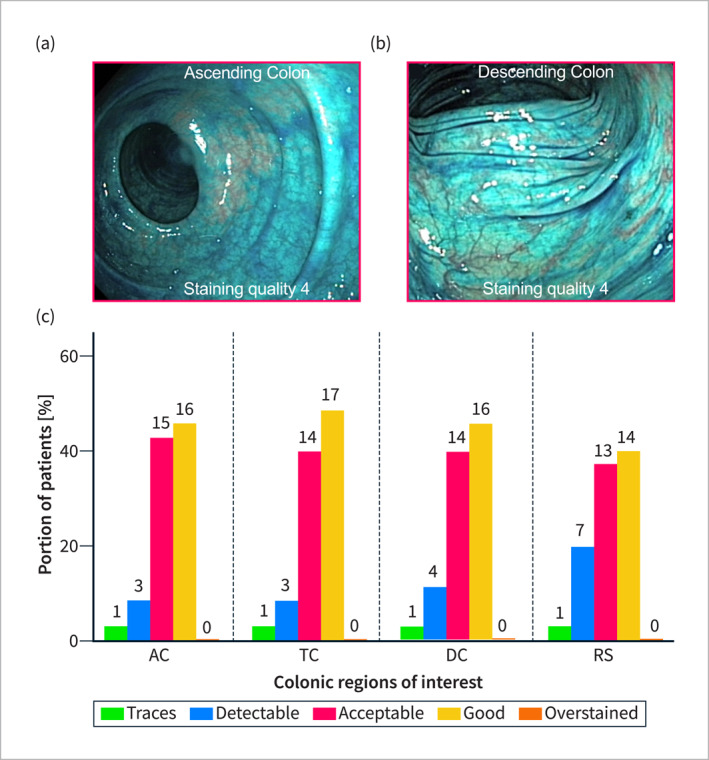
(a,b) Examples of colonic staining with SQ = 4 in the ascending and descending colon. (c) Comparison of staining quality per segment (AC = ascending colon; DC = descending colon; RS = recto‐sigmoid colon; TC = transverse colon).

**FIGURE 4:**
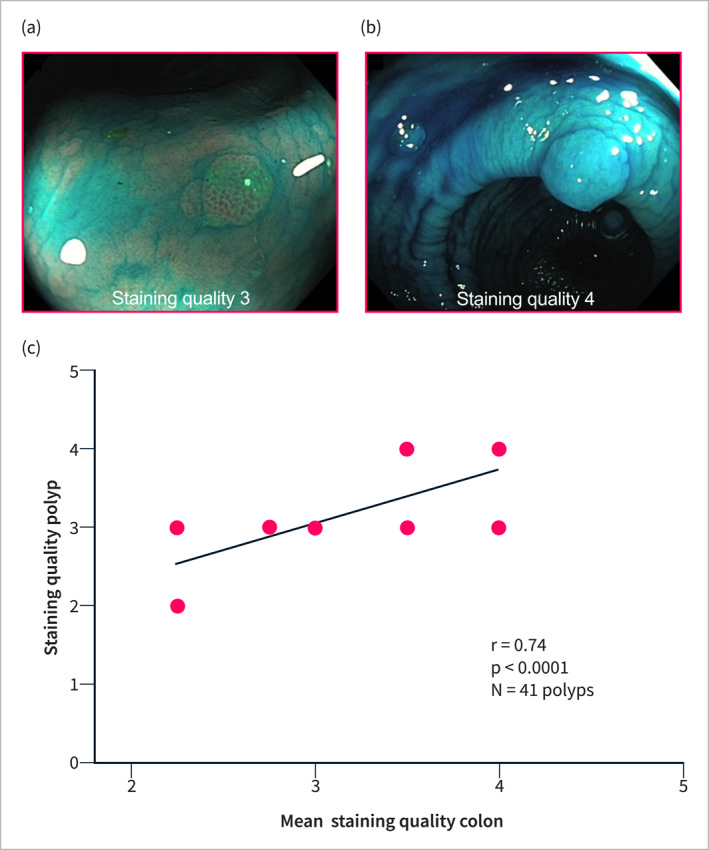
(a,b) Examples of polyp staining with SQ = 3 and SQ = 4. (c) Correlation analysis of mean staining quality of the colon and the respective staining quality of detected polyps. Due to an overlay of the correlated data, not all 41 polyps can be displayed.

In the PBM cohort, an optimal to acceptable SQ score of ≥ 8 was observed in 34/35 patients (97.1%), with a mean total score of 13.4 ± 2.9 (score range: 4–16). No patients showed overstaining (score = 5) in any segment. The SQ was generally consistent across all anatomical segments (SQ_AC_ = 3.4 ± 0.7; SQ_TC_ = 3.5 ± 0.7; SQ_DC_ = 3.4 ± 0.8; SQ_SR_ = 3.2 ± 0.9; *p* = 0.7 for all comparisons; Figure 3). Polypoid lesions were effectively stained, achieving a mean SQ score of 3.4 ± 0.6 (score range: 2–4). The SQ of the polyps showed a strong correlation with the individual mean staining score (*r* = 0.74, *p* < 0.0001; Figure 4).

The Cohen's kappa coefficient for interobserver agreement on SQ between the primary and secondary endoscopists was 0.87, reflecting a high level of consistency.

### 3.4 PDR/ADR

The PDR was significantly higher in the PBM group at 62.8% compared to 42.9% in the control group (*p* = 0.04). Similarly, the ADR showed a notable difference, with 45.7% in the PBM group versus 27.6% in the control group, although this did not reach statistical significance (*p* = 0.06). When evaluating polyps per colonoscopy, the PBM group exhibited a significantly higher mean (1.1 ± 1.1) compared to the control group (0.6 ± 0.8; *p* = 0.02). A similar pattern was observed for adenomas per colonoscopy, with the PBM group having a mean of 0.8 ± 0.9 versus 0.3 ± 0.5 in the control group (*p* = 0.02). The PBM group had a significantly higher percentage (41.2%) for medium‐sized polyps (6–10 mm) compared to the control group (15.3%; *p* = 0.003). No cases of colorectal carcinoma were reported in either group. Results including detailed distribution of the polyp sizes are summarized in Table 2.

**TABLE 2:** Comparison of detected polyps and adenomas per group.

	Total			Diagnostic colonoscopy 1:3 ratio propensity score matching			Surveillance in ulcerative colitis 1:3 ratio propensity score matching		
	Patent blue V microcapsules	Control	*p* value	Patent blue V microcapsules	Control	*p* value	Patent blue V microcapsules	Control	*p* value
Number of patients [*N*]	35	105	—	21	63	—	14	42	—
Polyp detection rate (*N*) [%]	22 (62.8)	45 (42.9)	0.04	11 (52.4)	26 (41.3)	0.5	11 (78.6)	19 (36.5)	0.04
Adenoma detection rate (*N*) [%]	16 (45.7)	29 (27.6)	0.06	9 (42.9)	16 (25.4)	0.2	7 (50)	13 (25)	0.2
Total number of polyps [*N*]	51	59	—	16	32		35	26	—
Polyps per colonoscopy [Mean ± SD]	1.1 ± 1.1	0.6 ± 0.8	0.02	0.8 ± 0.9	0.5 ± 0.7	0.1	1.4 ± 1.2	0.5 ± 0.9	0.01
Adenomas per colonoscopy [Mean ± SD]	0.8 ± 0.9	0.3 ± 0.5	0.02	0.7 ± 0.9	45	0.04	0.9 ± 1.1	0.3 ± 0.5	0.09
Polyp sizes [*N*] (% of all polyps)									
1–5 mm	19 (37.3)	29 (49.1)	0.25	8 (50)	15 (46.9)	> 0.9	11 (31.4)	14 (53.8)	0.1
6–10 mm	21 (41.2)	9 (15.3)	0.003	4 (25)	3 (9.4)	0.2	17 (48.6)	6 (23.1)	0.06
11–15 mm	9 (16.6)	19 (32.3)	0.1	3 (18.8)	14 (43.7)	0.1	6 (17.1)	5 (19.2)	> 0.9
> 15 mm	1 (1.9)	2 (3.4)	> 0.9	1 (6.2)	0	0.3	1 (2.9)	1 (3.8)	> 0.9
Incidence of colorectal carcinoma [%]	0	0	> 0.9	0	0	> 0.9	0	0	> 0.9

### 3.5 Adverse Events

The overall incidence of adverse events (AE) was similar between the two groups, with 12 AEs (34.3%) in the PBM group and 44 AEs (41.9%) in the control group (*p* = 0.5). All recorded AEs were classified as mild. The majority of AEs were related to gastrointestinal disorders (PBM: 4 (11.4%), control: 14 (13.3%), *p* > 0.9). Specific gastrointestinal symptoms included abdominal discomfort (PBM: 2 (5.7%), control: 6 (5.7%), *p* > 0.9) and nausea (PBM: 2 (5.7%), control: 7 (6.7%), *p* > 0.9). Vomiting was rare (PBM: 0, control: 1 (0.9%), *p* > 0.9). No intervention‐related complications were recorded (Table S2).

### 3.6 Post Hoc Power Analysis

A post hoc power analysis was performed to assess the statistical power of the study. The primary endpoint was the proportion of patients achieving an acceptable to optimal SQ that was achieved by 97.1% (34/35) of patients. With a non‐inferiority margin of 0.1% and a one‐sided significance level of *α* = 0.05, the calculated post hoc power was 1.1%. This result confirms that the study is significantly underpowered to clearly demonstrate non‐inferiority.

## 4 Discussion

Chromoendoscopy plays a vital role in both diagnostic colonoscopy for patients with positive FOBT and surveillance colonoscopy for patients with long‐standing UC.

In the context of patients who present with positive FOBT results, PDR and ADR serve as primary benchmarks for the effectiveness of the procedure [1]. Several technical options to improve the PDR/ADR in diagnostic colonoscopy have already been compared with HDWLE. Real dye‐based chromoendoscopy using a spray catheter has been the gold standard for a long time [22]. In this regard, a risk ratio of 1.16 between HDWLE and dye‐based chromocolonoscopy was reported in a recent meta‐analysis [3]. The administration of dyes to the colonic surface can be categorized into selective and unselective techniques. Selective application targets specific lesions identified initially via white‐light imaging (WLI), whereas unselective application involves broad coverage of the mucosa to enhance lesion detectability. The efficacy of unselective, pan‐chromoendoscopy remains inconclusive; a notable improvement in both PDR and ADR, particularly in the right hemicolon, suggests a potential benefit [5, 23]. Other investigations have not demonstrated significant advantages over standard colonoscopy or HDWLE [24, 25]. On the contrary, chromoendoscopy is valuable for differentiating neoplastic from non‐neoplastic lesions, delineating their margins, assessing surface characteristics and estimating the risk of deeper mucosal invasion [26].

Chromoendoscopy remains a cornerstone in surveillance colonoscopy for patients with long‐standing UC, where the risk of colorectal cancer arises through an inflammation‐dysplasia‐carcinoma sequence. In this context, dye‐based chromoendoscopy improves the detection of subtle, flat dysplastic lesions that are often missed by standard white‐light endoscopy. Numerous studies have demonstrated that dye spray techniques, including the use of methylene blue or indigo carmine, significantly increase dysplasia detection rates in UC patients compared to conventional random biopsies [27, 28]. Reflecting this evidence, both the SCENIC consensus [29] and the European Crohn and Colitis Organization (ECCO) guidelines [7] recommend chromoendoscopy with targeted biopsies as the preferred surveillance approach in UC. While virtual chromoendoscopy techniques such as NBI offer advantages in convenience and enhanced vascular pattern recognition, they have not consistently demonstrated dysplasia detection rates equivalent to dye‐based methods, particularly in the context of chronic inflammation [30, 31]. The present study focused on staining quality as a fundamental performance marker in patients with UC, recognizing that effective mucosal contrast is essential for optimizing dysplasia detection [32].

Despite the proven advantages of chromoendoscopy, a widespread adoption is limited by the cumbersome nature of the dye‐spray technique, requiring additional time for the procedure and sedation as well as extensive cleaning of the equipment.

In contrast, the newly developed PBM formulation offers a simplified and patient‐friendly approach to mucosal staining. In this proof‐of‐concept study, PBM yielded adequate SQ across all colonic segments, with no evidence of overstaining or unstained areas, in both patient groups. These results support the feasibility and effectiveness of this novel PBM formulation in enhancing mucosal contrast. The favorable staining performance, coupled with the ease of administration, suggests that the capsule‐based approach may improve both patient comfort and procedural quality in clinical practice.

To date, only one formulation with a comparable mode of application has received market approval (Methylene blue MMX (MB‐MMX)). In a multicenter Phase III trial, homogeneous SQ was achieved in over 90% of patients, accompanied by a significantly higher ADR in the MB‐MMX group (56.29%) compared to placebo (47.81%) [8]. These findings are partially consistent with our own results. We observed a significant increase in the number of polyps and adenomas detected per colonoscopy, primarily driven by a higher detection rate of polyps measuring 6–10 mm in diameter. The PDR in our cohort was 62.8%, which was significantly higher than the 42.9% observed in the control group (*p* = 0.04). Although the ADR was also notably higher in the intervention group (45.7% vs. 27.6%), this difference did not reach statistical significance (*p* = 0.06), likely due to the limited sample size and insufficient statistical power of our proof of concept design. In addition to the trial with MB‐MMX [8], our study demonstrated adequate staining quality in patients with UC.

While MB‐MMX has received regulatory approval following clinical safety evaluations, some preclinical studies have reported that MB may induce DNA damage in colonocytes through oxidative stress mechanisms [10]. Although no clinically relevant AE related to DNA damage has been observed in routine use, it may still be prudent to consider minimizing cumulative exposure to agents with potential genotoxic effects—particularly in high‐risk populations such as patients with UC who may require repeated endoscopies. In contrast, the EFSA approved PB for use in various food products. Extensive evaluations have shown that PB is practically not absorbed into cells, shows only a minimal systemic exposure [33] and no genotoxic potential, supporting its safety as a food additive [12] and reliable choice for chromocolonoscopy.

With the advent of emerging technologies, the clinical role of chromocolonoscopy may diminish in the future. This trend is supported by a recent network meta‐analysis including over 60,000 patients from 94 randomized controlled trials, which evaluated the impact of various endoscopic visualization techniques on ADR [3]. The analysis revealed that newly developed artificial intelligence (AI) algorithms significantly improve ADR compared to traditional methods such as dye‐based chromoendoscopy. However, serrated adenomas are a significant contributor to the development of interval colorectal cancers. These lesions often elude identification by AI algorithms, which typically categorize them as non‐adenomatous. This misclassification can mislead endoscopists, given the high transformation risk of these lesions into colorectal cancer. Currently, chromocolonoscopy remains the gold standard for differentiating this particular subgroup of serrated adenomas [34]. Looking ahead, the integration of AI with advanced chromocolonoscopy could enhance diagnostic accuracy by leveraging the contrast enhancement of chromoendoscopy with the pattern recognition capabilities of AI. In this context, capsule‐based chromoendoscopy may find its most appropriate clinical application in clearly defined patient populations. These include: (i) individuals undergoing diagnostic or screening colonoscopy in whom serrated or atypical adenomas are suspected and require improved mucosal visualization and classification and (ii) patients with long‐standing UC undergoing dysplasia surveillance, where enhanced mucosal contrast is critical for detecting flat or subtle lesions.

### 4.1 Limitations

A significant limitation of this case series is the small sample size of 35 patients with a calculated power of only 1.1%. While patients were prospectively enrolled under “compassionate use” conditions as a proof concept, this work did not employ randomization or blinding to the respective study groups. Furthermore, this proof‐of‐concept case series did not include a direct control arm using the dye‐spray technique, nor were the endoscopists blinded to the type of staining performed.

## 5 Conclusion

In conclusion, this proof‐of‐concept demonstrated that orally administered PBM effectively stained the colonic mucosa in patients undergoing diagnostic and surveillance colonoscopy. The use of PBM was associated with a significant improvement in PDR and a notable increase in ADR. Further randomized controlled trials are warranted to validate these findings and to compare PBM with conventional dye‐spray chromoendoscopy, particularly in combination with emerging AI‐based detection algorithms.

## Ethics Statement

The evaluation of this case series received approval from the Ethics Committee of the Medical Faculty at Christian‐Albrechts‐University, Kiel, Germany (Reference Numbers: D439/15 and D579/23). Written informed consent was obtained from all participants.

## Conflicts of Interest

Patent blue V microcapsules are protected by the European patent no. 3445336 (authors among the inventors, in alphabetical order: M. Ellrichmann, J. K. Keppler, S. Schreiber, K. Schwarz, E.‐M. Theismann, and G. H. Waetzig). G. H. Waetzig is partially employed by CONARIS Research Institute AG (Kiel, Germany). M. Ellrichmann received consultation fees from Olympus and Medtronic. All other authors have nothing to disclose.

## Supporting information

Table S1

Table S2

## Data Availability

The data that support the findings of this study are available from the corresponding author upon reasonable request.
