# Supplementary material for: Oral Microcapsule Chromocolonoscopy With Patent Blue V Improves Adenoma Detection Safely and Effectively
Source: United European Gastroenterol J. 2025 Jul 5;13(7):1116–26. doi: 10.1002/ueg2.70067 (PMC12463709; doi:10.1002/ueg2.70067)
Supplement: Supplementary file 1 — Table S1 [file UEG2-13-1116-s002.docx]

**Schulte B, Waetzig GH *et al.*: Oral Microcapsule Chromocolonoscopy With Patent Blue V Improves Adenoma Detection Safely and Effectively.**

**Supporting Information**

**Table S1:** Protocol of the bowel preparation and capsule intake

| **Day 1** | |
| --- | --- |
| 5 pm | 1 L KleanPrep |
| 6 pm | 1 L KleanPrep followed by 2 capsules |
| 7 pm | 1 L KleanPrep followed by 2 capsules |
| 8 pm | Still water |
| **Day 2** | |
| 6 am | 1 L KleanPrep followed by 2 capsules |
| 7 am | Still water |
| from 11 am | Colonoscopy |
