# Supplementary material for: Oral Microcapsule Chromocolonoscopy With Patent Blue V Improves Adenoma Detection Safely and Effectively
Source: United European Gastroenterol J. 2025 Jul 5;13(7):1116–26. doi: 10.1002/ueg2.70067 (PMC12463709; doi:10.1002/ueg2.70067)
Supplement: Supplementary file 2 — Table S2 [file UEG2-13-1116-s001.docx]

**Schulte B, Waetzig GH *et al.*: Oral Microcapsule Chromocolonoscopy With Patent Blue V Improves Adenoma Detection Safely and Effectively.**

**Supporting Information**

**Table S2:** Overview of adverse events

|  | **Patent blue V microcapsules (PBM)** | **Control** | ***p* value** |
| --- | --- | --- | --- |
| **All adverse events [N] (%)** | 12 (34.3) | 44 (41.9) | 0.5 |
| mild [N] (%) | 12 (34.3) | 44 (41.9) | 0.5 |
| moderate [N] (%) | 0 | 0 | > 0.9 |
| severe [N] (%) | 0 | 0 | > 0.9 |
| Gastrointestinal disorders [N] (%) | 4 (11.4) | 14 (13.3) | > 0.9 |
| Abdominal discomfort [N] (%) | 2 (5.7) | 6 (5.7) | > 0.9 |
| Haematemesis [N] (%) | 0 | 0 | > 0.9 |
| Gastrointestinal haemorrhage [N] (%) | 0 | 0 | > 0.9 |
| Melaena [N] (%) | 0 | 0 | > 0.9 |
| Nausea [N] (%) | 2 (5.7) | 7 (6.7) | > 0.9 |
| Vomiting [N] (%) | 0 | 1 (0.9) | > 0.9 |
| Urinary tract signs and symptoms [N] (%) | 1 (2.9) | 3 (2.9) | > 0.9 |
| Polyuria [N] (%) | 1 (2.9) | 3 (2.9) | > 0.9 |
| Nervous system disorders [N] (%) | 1 (2.9) | 5 (4.8) | > 0.9 |
| Headache [N] (%) | 1 (2.9) | 4 (3.8) | > 0.9 |
| Migraine [N] (%) | 0 | 1 (0.9) | > 0.9 |
| Blood and lymphatic system disorders [N] (%) | 0 | 0 | > 0.9 |
| Anaemia [N] (%) | 0 | 0 | > 0.9 |
| Haemorrhagic anaemia [N] (%) | 0 | 0 | > 0.9 |
| General disorders [N] (%) | 0 | 0 | > 0.9 |
| Intervention related complications [N] (%) | 0 | 0 | > 0.9 |
| mild [N] (%) | 0 | 0 | > 0.9 |
| moderate [N] (%) | 0 | 0 | > 0.9 |
| severe [N] (%) | 0 | 0 | > 0.9 |
